# Supplementary material for: Trapping/Pinning of colloidal microspheres over glass substrate using surface features
Source: Sci Rep. 2017 Nov 16;7:15754. doi: 10.1038/s41598-017-15984-4 (PMC5691049; doi:10.1038/s41598-017-15984-4)
Supplement: Supplementary file 1 — Supplementary Information [file 41598_2017_15984_MOESM1_ESM.pdf]

Supplementary Information

Trapping/Pinning of colloidal microspheres over glass substrate using surface features

Praneet Prakash[1], Manoj Varma[1,2]

[1]Center for Nano Science and Engneering, Indian Institute of Science, Bangalore

[2]Robert Bosch Center for Cyber Physical Systems, Indian Institute of Science, Bangalore

[*]mvarma@iisc.ac.in

Explanation of the real time video shown in the movie " $supple\_pinning1.mov$ ".

1. ($t = 0 - 31\ sec$): Pinning of 10 μm microspheres shown in Fig. 5(a) near the microstructure of $h = 1.1\ \mu m$, $w = 5.1\ \mu m$ (Fig. 3(a)). Initial flow rate is 50 $\mu l/min$, however, pinning starts only when the flow rate is reduced to 10 $\mu l/min$.

2. ($t = 32 - 43\ sec$): Dark field video of 5 μm pinned microspheres shown in Fig. 5(b) near the microstructure of $h = 1.1\ \mu m$, $w = 5.1\ \mu m$ (Fig. 3(a)).

3. ($t = 44 - 1{:}44\ sec$): Trapping/pinning of 10 μm microspheres over the downslope of the microstructure of $h = 4.3\ \mu m$, $w = 19.2\ \mu m$ (Fig. 3(c)) at a high flow rate of 50 $\mu l/min$ which is shown in Fig. 7(a) & 7(b).

4. ($t = 1{:}44 - 2{:}14\ sec$): Pinning is not observed at a high flow rate of 50 $\mu l/min$ on the microstructure of $h = 4.1\ \mu m$, $w = 5.4\ \mu m$ (Fig. 3(b)) unless the pump is stopped.

Explanation of the real time video shown in the movie " $supple\_pinning2.mov$ ".

1. ($t = 0 - 15\ sec$): Pinning in the upslope region at a low flow rate of 0.1 $\mu l/min$ on the microstructure of $h = 1.1\ \mu m$, $w = 5.1\ \mu m$ (Fig. 3(a)).

2. ($t = 15 - 32\ sec$): Pinning in the downslope region at a low flow rate of 0.1 $\mu l/min$ on the microstructure of $h = 4.1\ \mu m$, $w = 5.4\ \mu m$ (Fig. 3(b)).

Explanation of the real time video shown in the movie " $supple\_pinning3.mov$ ".

This video shows the switching of pinned microspheres from the upslope region to the downslope region as the flow rate is increased. Initially microspheres were pinned in the upslope region of the microstructure ($h = 4.1\ \mu m$, $w = 5.4\ \mu m$) at a low flow rate of $0.1\ \mu l/min$, as the flow rate is increased to $30\ \mu l/min$ they switch to downslope region. Hence, the hydrodynamic interaction has an important role in facilitating robust pinning in the downslope region at higher flow rates.

Comparison of drag force calculated from simulation and analytical formula

The drag force mentioned in the manuscript corresponds to the steady state solution, where microspheres are assumed to be pinned and a no-slip boundary condition is applied on the microfluidic channel as well as the microsphere. The accuracy of the calculated drag force by simulation can be confirmed by assuming the microsphere at the centre which will result in a drag force $F_d = 6\pi\eta r v$, where, η, r are viscosity, radius of the sphere and v is the velocity of the approaching fluid far from the microsphere. At a flow rate of $60\ \mu l/min$ analytically calculated drag force $F_{d(analytical)} = 3.09\ nN$ which is very close to the drag force

calculated from the simulation $F_{d(simulation)} = 3.12\, nN$. Further, the estimation of the drag force at the bottom of the microfluidic channel is also in accordance with the analytical expression used in previous reports $F_{d(bottom)} = 1.7 \times 6\pi\eta r v$ (Reference: 22). The analytically calculated drag force at the bottom of the channel (flow rate of $60\, \mu l/min$) for a microsphere of radius $5\, \mu m$ is $F_{d(analytical)} = 0.977\ nN$ which is very close to the drag force estimated from the simulation $F_{d(simulation)} = 0.86\, nN$.

Estimation of DLVO force between microspheres and the glass substrate

The van der Waals force between a spherical object and a flat surface is $F_V = -AR/6D^2$, where, A is Hamaker constant and R & D are the radius of microsphere and its distance from the substrate respectively. We have used commonly available glass slide as a substrate which contains sodium and calcium silicates (Na_2Sio_3, Ca_2Sio_4) along with fused silica ($amorphous\, Sio_2$). We couldn't find the Hamaker constant of plystyrene-water-glass system, however, for polystyrene-water-polystyrene system it comes out to be $0.95 - 1.3 \times 10^{-20}\, J$. Hamaker constant for other silica based solids such as fused quartz and mica in water comes out to be $\sim 0.5 - 3 \times 10^{-20}\, J$ (Ref. 33 – Intermolecular and Surface Forces, 3rd Edition, Jacob N. Israelchvili, Page No. 266). Considering the value of Hamaker constant to be $1 \times 10^{-20}\, J$ and the radius of microsphere and its distance from the substrate as $5\, \mu m$ & $0.2\, nm$ respectively, the van der Waals force comes out to be $-206.25\, nN$. Typically the distance between two surface in contact is ~ 0.2 nm (Ref. 33 – Intermolecular and Surface Forces, 3rd Edition, Jacob N. Israelchvili, Page No. 254), the respective $F_v = -206.25\, nN$ is much larger than the fluidic force generated by the flow in the pinning regime which is below $1\, nN$. The calculation of the electrostatic force F_E requires the estimation of the Zeta potential of the polystyrene microsphere and glass substrate. The polystyrene microsphere has a Zeta potential of $\sim -40\, mV$ in $0.01\, M$ PBS buffer at a pH of 7.5 (Ref. 34). We were not able to find the Zeta potential of glass substrate in PBS buffer, hence, we have used Zeta potential of fused silica ($amorphous\, Sio_2$) $\sim -60\, mV$ in $0.01\, M$ KCl solution at a pH of 7.5 (Ref. 35). The range of the electrostatic force depends upon the screening of charges by the ions present in the medium which is defined by the Debye length. The Debye length l_D for a low potential surface (below $\sim 25\, mV$) in a 2:2 electrolyte system such as $MgSo_4$ is defined by the expression $0.152/\sqrt{(C)}\ nm$, where C is the concentration of salt solution in $Molar$ (Ref. 33 – Intermolecular and Surface Forces, 3rd Edition, Jacob N. Israelchvili, Page No. 312). We have done all our experiment in $0.01\, M$ PBS buffer, the major constituent of the buffer being Na_2HPO_4, which is a 3:3 electrolyte. For an order of calculation estimate, we find the Debye length by using $0.152/\sqrt{(C)}\ nm$ which comes out to be 1.5 nm, for $C = 0.01\, M$. The electrostatic force between a microsphere and a flat surface of similar potential is $F_E = 2\pi R W_{flat}$, where, R is the radius of microsphere and the interaction free energy $W_{flat} = 0.0211\, (C)^{1/2} tanh^2[2\varphi(mV)/103] e^{-D/l_d}\, Jm^{-2}$. The electrostatic force (repulsive) F_E at a contact distance of $0.2\, nm$ (Ref. 33 – Intermolecular and Surface Forces, 3rd Edition, Jacob N. Israelchvili, Page No. 254), is $34.3\, nN$ whereas van der Waals force (attractive) is $-206.25\, nN$, hence, the van der Waals force dominates in the sub-nanometre length scale.

Table 1 Parameters for wet etching.

Mirostructure	Photoresist	Resist width	Periodicity	Post-bake time	HF etch duration
$h = 1.1\ \mu m, w = 5.1\ \mu m$	S1813	$5\ \mu m$	$50\ \mu m$	3 min @ 110°C	$10 - 15\ sec$
$h = 4.1\ \mu m, w = 5.4\ \mu m$	AZ4562	$5\ \mu m$	$50\ \mu m$	5 min @ 110°C	$40 - 45\ sec$
$h = 4.3\ \mu m, w = 19.2\ \mu m$	AZ4562	$20\ \mu m$	$100\ \mu m$	5 min @ 110°C	$40 - 45\ sec$

Figure S1

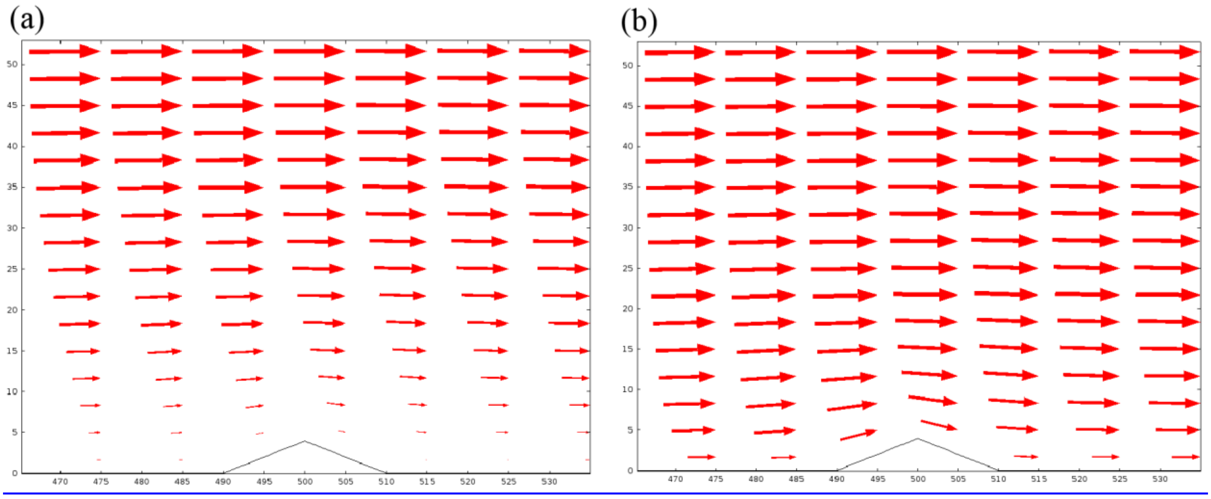

Fig. S1 Velocity vector field over the surface of microstructure ($h = 4\ \mu m,\ w = 20\ \mu m$) at a volumetric flow rate of $50\mu l/min$. Here we have shown only half of the channel which is from the bottom to the middle. (a) Arrow of the velocity vector field is proportional to the magnitude of the velocity with a maximum velocity of $2.5\ cm/s$ at the top of the graph. (b) Arrow of the velocity vector field is proportional to the logarithmic of the magnitude of velocity so that the velocity vector arrows which are very near to the bottom surface are also visible.

Figure S2

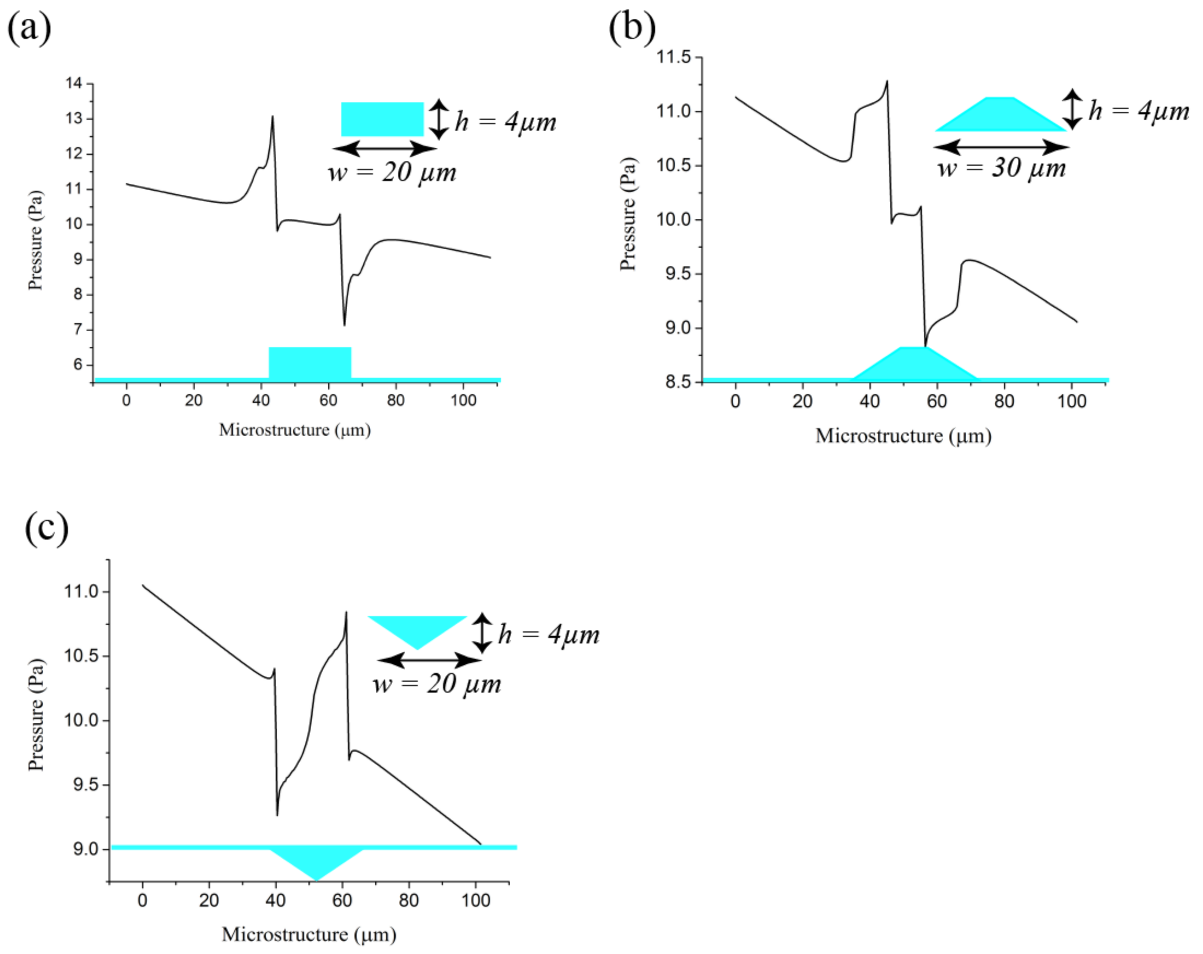

Fig. S2 Pressure profile plots over the surface of microstructure with different geometries. Volumetric flow rate was $50\mu l/min$ and the channel domain was taken to be $1\,mm$ long. (a) Square (b) Trapezoid (c) Triangular shaped trough.

Fabrication of a square shaped crests isn't possible with isotropic wet etch, however, Deep reactive-ion etching can be employed to fabricate them. Trough structures can be explored to see their pinning ability, however, microspheres should be very close to the substrate surface for it to interact with the lower regions and hence, random pinning may increase.

Figure S3

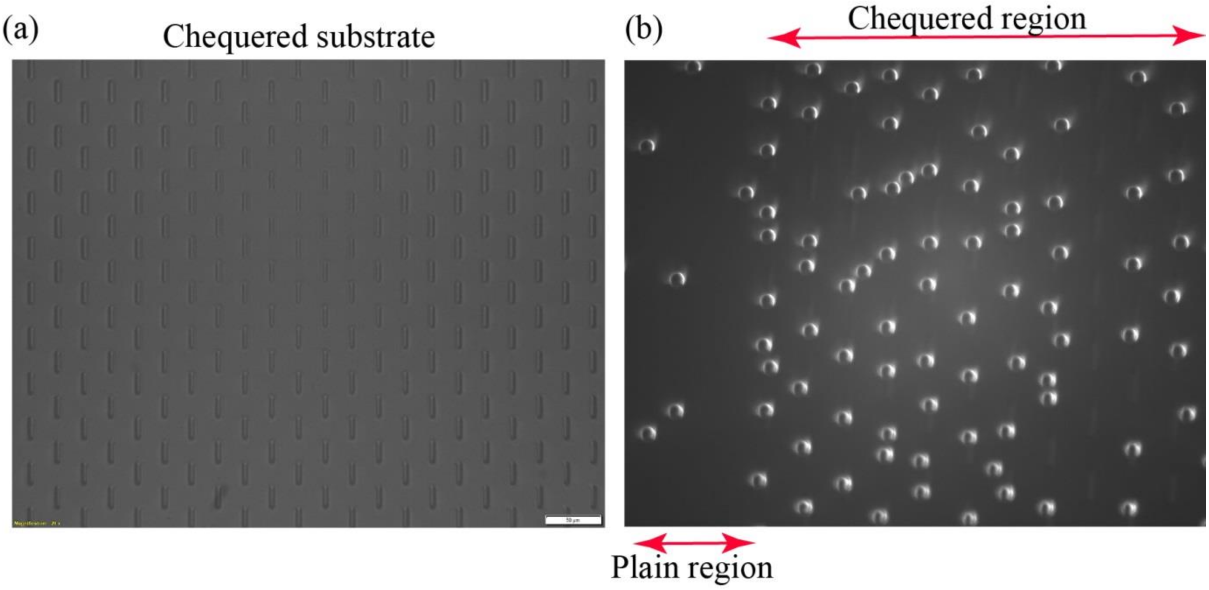

Fig. S3 Pinning over a chequered substrate. (a) A chequered substrate with alternate microstructures of $h = 1.1\ \mu m,\ w = 6.5\ \mu m$. (b) Chequered pattern of microspheres enabled by the positioning of microstructures.

A substrate such as shown above can enable the precise positioning of microspheres according to the pattern of microstructure.
